# The use of religious resources in helping victims of workplace mobbing

**DOI:** 10.3389/fpsyg.2023.1288354

**Published:** 2023-11-17

**Authors:** Jolita Vveinhardt, Mykolas Deikus

**Affiliations:** ^1^Vytautas Kavolis Transdisciplinary Institute for Social Sciences and Humanities, Vytautas Magnus University, Kaunas, Lithuania; ^2^Department of Management, Faculty of Economics and Management, Vytautas Magnus University, Kaunas, Lithuania; ^3^Department of Theology, Faculty of Catholic Theology, Vytautas Magnus University, Kaunas, Lithuania

**Keywords:** workplace mobbing, victim, consequences, assistance, religiosity, Christian spiritual assistance

## Abstract

Research shows that in providing assistance to individuals who have experienced psychological traumas, it is beneficial not only to take into account the specific religious spiritual needs but also to employ religious resources. Although the role of religious counsellors using various psychological theories in helping to cope with traumatising experiences is acknowledged, there is still a lack of a conceptualising approach to the possibilities of employing religious resources used in Christian spiritual assistance, seeking to help cope with the effects of workplace mobbing. Therefore, this study aims to conceptualise the perspective of integrating Christian spiritual assistance resources in overcoming the individual consequences of workplace mobbing. This review is based on an interdisciplinary approach and abundant literature of psychology, psychotherapy and theology sciences. The article details the main physical, psychological and social aspects of damage to the person, caused by mobbing, which provide for a corresponding triple assistance perspective. After highlighting the essential resources provided by the Christian religion for coping with traumatic experiences, the necessity to consider the religious needs of the victims of mobbing is reasoned and the possibilities of using religious spiritual resources are discussed. In the context of helping victims of mobbing, two main functions of Christian spiritual assistance are distinguished and discussed: auxiliary and main. The results of this review are a useful resource for lay and religious counsellors and encourage their collaboration. The research findings also provide a basis for further research on the use of religious resources in the context of helping victims of mobbing.

## 1 Introduction

According to psychiatrist M. Scott Peck, quoted by Davenport et al. in the book “Mobbing: Emotional Abuse in the American Workplace,” evil is a force that resides both inside and outside the person. Evil people use force, wishing to destroy the spiritual growth of others in order to defend and preserve their own disturbed integrity (Davenport et al., 1999: 59). The question of why the innocent suffer from evil has always concerned people. Not only the search for the answer but also the psychological states experienced when evil invades one’s life were aptly described in the Old Testament Book of Job as early as some two and a half thousand years ago. It highlights experiences such as anxiety, insomnia, the pain caused by abandonment and injustice, existential emptiness and helplessness (*cf.* Job 7, 3–4, 6), which essentially describes what the victims of mobbing also experience [e.g., anxiety and insomnia (Vartia, 2001), helplessness (Krishna et al., 2023), etc.].

Workplace mobbing refers to incidents such as verbal harassment, aggressive words, sarcasm, slander or social isolation directed at a specific person (Tatar and Yüksel, 2019). This phenomenon differs from other forms of harmful behaviour in the workplace in that assaults last for a long period of time, are systematically repeated, and more than one co-worker is involved in the harassment of the individual (Leymann, 1996; Nielsen et al., 2015; Pheko, 2018; Tatar and Yüksel, 2019). The term “bullying” is also used in the literature to refer to this phenomenon. According to Leymann (1996), the connotation of “bullying” is related to “physical aggression and treat,” while workplace violence manifests itself more subtly (e.g., by social isolation). He therefore proposed that the term “bullying” should be retained to describe the behaviour of pupils. Meanwhile, Einarsen et al. (2011) believe that the terms bullying and mobbing can be used interchangeably. However, it has to be considered that the term “bullying” is used not only to describe the relationships between employees but also between children and pupils. Therefore, in order to avoid confusion, the term “mobbing” proposed by Leymann is further followed in the cases under consideration, when the person becomes the target of long-term and systematically repeated unwanted behaviour of colleagues. All of this is accompanied by severe prolonged stress, which the target is usually unable to cope with (Leymann, 1990; Lee and Lim, 2019). Regardless of which term is used, studies emphasize severe consequences for the target, such as PTSD (Nielsen et al., 2015; Tatar and Yüksel, 2019) or burnout (Karsavuran and Kaya, 2017; Aristidou et al., 2020).

Although traditionally the largest scholarship on workplace mobbing studies is associated with such scientific fields as psychology, law, sociology and management, a broader interdisciplinary approach, which would be useful for a deeper understanding of the phenomenon, is still needed (Fahie and Dunne, 2021). In this context, it is significant that a quarter of a century ago the World Health Organization (WHO) acknowledged the importance of an individual’s spiritual needs and religious beliefs for health and quality of life (World Health Organization, 1998) and that further research has confirmed that religiosity helps to cope with stress, fear, and anxiety (Prazeres et al., 2021), to overcome depression (Bakır et al., 2021) and psychological trauma accompanied by post-traumatic stress disorder (Harris et al., 2021). However, despite the existing evidence, religious spiritual assistance for the victims of mobbing, which responds to the person’s specific needs, remains under-researched in the scientific literature, both theoretically and empirically (Vveinhardt and Deikus, 2022a).

A study conducted more than 10 years ago by Vensel (2012) demonstrated that some Christian spiritual practices helped in coping with burnout, which could also be a consequence of workplace mobbing. Still another study states that workplace chaplains are also approached when experiencing bullying, emotional or mental health problems (Gilliat-Ray et al., 2023). Thus, although indirectly, this demonstrates that Christian resources can be useful for employees affected by mobbing; however, episodic excursions into this painful topic do not cover the possibilities of applying Christian spiritual assistance strategies to help victims. Effective application of Christian spiritual help to the victims of mobbing requires broader research of conceptualizing nature, modelling various assistance strategies. Therefore, this study aims to conceptualise the perspective of integrating Christian spiritual assistance resources in coping with individual consequences of workplace mobbing.

This paper analyses individual consequences of mobbing for the victims of mobbing and how these consequences can be facilitated by integrating religious resources in practice. The study is based on an interdisciplinary approach, reviewing the literature in psychology, psychotherapy and theology. Its findings broaden the understanding of assistance to the victims of mobbing, accentuate the role of religious faith and provide a basis for further empirical research on Christian spiritual assistance for the victims of mobbing.

## 2 Methods

A narrative approach was applied, which is valuable “when one is attempting to link together many studies on different topics, either for purposes of reinterpretation or interconnection” (Baumeister and Leary, 1997, 312). A narrative literature review is used to expand understanding in a certain area (Greenhalgh et al., 2018) and highlights key findings about the phenomenon rather than provides a systematic assessment of the research area (Nielsen and Einarsen, 2018). This does not necessarily involve a systematic literature search strategy (Snyder, 2019); therefore, in response to the criticism that “random evidence” may be taken, Greenhalgh et al. (2018) emphasize that producers of narrative reviews gather evidence in a deliberate and purposeful way, considering what is important in addressing key issues (Greenhalgh et al., 2018).

A search for conceptual and empirical research was carried out in the Web of Science, SCOPUS, PubMed, ProQuest databases, with an additional search in Google Scholar. The search, which was limited to years 1990–2023, was performed using various combinations of keywords such as “workplace mobbing,” “workplace bullying,” “individual consequences,” “Christian,” “spirituality,” “support,” “assistance,” “counseling,” “pastoral care.” Studies using the keywords “mobbing” or “bullying” were included in cases where they corresponded to the definition given in the introduction of this article (taking into account the systematicity and duration of actions). When examining the religious aspect of assistance, we have included works on practices used in the Christian religion. In addition, the main focus was on articles that appeared first and were cited more often.

## 3 Areas of personal life, affected by mobbing

The archaic mechanism of group persecution, which Girard (1986) identifies as scapegoating, emerges in the workplace as a unique and particularly harmful form of aggression in the workplace (Nielsen and Einarsen, 2018). Compared to the outbursts of physical violence, which occur when a crowd persecutes a “perpetrator,” workplace violence manifests itself in more subtle forms (for more see Leymann, 1996). However, the behavior of persecutors in a group means that the force of the violent act “is increased with the addition of each individual quotient of violence” (Girard, 2005, 35), while systematic, long-lasting hostile and offensive behavior at work leads to various negative health consequences, including somatic and psychological symptoms (Nielsen et al., 2015).

On the one hand, research shows that, depending on the type, time and intensity of the irritant, stress can cause physiological changes ranging from homeostatic changes to life-threatening health disorders and death (Yaribeygi et al., 2017). On the other hand, the experience of mobbing stands out from the stress that is often experienced in various situations by its traumatic multifaceted nature. Therefore, Nielsen et al. (2015), in a meta-analysis of research on mobbing and post-traumatic stress disorder, highlighted not only worsened physical health but also persistent tension, burnout and psychological suffering experienced by both adult and adolescent victims of mobbing. The physical and psychological suffering experienced by victims according to aspects and noticeable signs is detailed in Table 1.

**Table 1:** Aspects of consequences of mobbing for the person.

Aspects	Disorders	Kind of research	Source
Physical	Sleep disorders	Qualitative research and systematic review	Vartia (2001) and Nielsen et al. (2020)
	Constant fatigue, nervousness	Quantitative research	Vartia (2001)
	Changes in attention span, body weight (increase or decrease)	Overview of research	Davenport et al. (1999)
	Disability	Overview of research	Hogh et al. (2019)
	Eczema	Case study	Duffy and Sperry (2007)
	Digestive disorders, high blood pressure	Overview of research	Davenport et al. (1999)
Psychological	Avoidance, intrusion, hypersensitivity	Meta-analysis of PTSD symptoms after mobbing experience	Nielsen et al. (2015)
	Suicidal ideation	Case study and quantitative research	Duffy and Sperry (2007) and Vartia (2001)
	Post-traumatic stress disorder	Overview of research and meta-analysis	Leymann (1996); Nielsen et al. (2015)
	Distress, tension, fear.	Quantitative research	Vartia (2001)
	Self-doubt, shame and humiliation leading to increased self-isolation from the natural social relations network, depression.	Case study and quantitative research	Duffy and Sperry (2007) and Vartia (2001)
Social	Social isolation, stigmatizing, voluntary unemployment, social maladjustment	Case study	Leymann (1990)
	Adversely affects family relationships	Empirical research	Machado et al. (2021)

It should be noted that division of consequences into physical and psychological is rather conditional, since the impact on the individual is not merely psychological and goes beyond personal suffering. On the one hand, the experienced attacks and social isolation cause spiritual pain for the person, while physical ailments are often psychosomatic in origin (de Pedro et al., 2008). That is, disorders of the human body manifest themselves as physiological reactions to prolonged and severe stress.

On the other hand, the victim’s social ties are disrupted, which causes suffering to both the victim and the surrounding people. Research shows that the person experiencing mobbing transfers negative emotions to the family; therefore, quite often these families experience conflicts, relationship breakdowns or eventually break up (Duffy and Sperry, 2007; Hillard, 2009). This is influenced by change in the victim’s behaviour, health disorders, and deteriorating material situation or abuse of alcohol, psychotropic drugs, drugs used when trying to cope with the crisis. Loved ones and friends see the change in the suffering person’s behaviour but do not always understand what is happening (Davenport et al., 1999). In addition, although mobbing influences deteriorating marital relations (alienation, conflicts, changes in sexual behaviour), the family also plays a protective role (Pellegrini et al., 2018).

The consequences of mobbing go beyond the clearly visible and felt physiological and psychological reactions. Some research on bullying among school-age young people show that a part of former victims later become abusers (Rivers and Noret, 2010). Thus, a certain process of learning dysfunctional social relationships takes place, which also allows to treat the bully as an injured person who needs help. Violence and suffering as a visible event therefore have deeper roots, going back to the past injuries of both the perpetrator and the victim (Twemlow et al., 1996).

## 4 Directions for assistance to persons who have experienced mobbing

Research shows that as the process of mobbing continues, the feelings of helplessness and disappointment arise, since attempts to defend oneself do not produce the expected results (Leymann, 1990; Pheko, 2018; Krishna et al., 2023). This means that the person becomes unable to overcome the situation on his own. Although it is important that opportunities should be ensured for affected employees to consult with the organisation’s internal and external professionals, receive long-term support, legal assistance or rehabilitation (Chappel and Di Martino, 2006), emotional support is equally significant. According to Mhaka-Mutepfa and Rampa (2021), emotional support and encouragement provided by co-workers is invaluable, as it can save the persecuted person’s life and career. It gives strength to start using existing assistance resources.

Although the problem of mobbing should first be tackled within the organisation itself, employing special preventive and intervention measures, in practice, the protection of employees is not always adequately ensured (Davenport et al., 1999; Turner, 2018). Since the victim is involved in the dangerous process gradually and does not always notice the first signs, opportunities of making rational decisions are sharply declining in the later stages of the process, when the damage has already been done (Leymann, 1996; Davenport et al., 1999). In addition, the victim needs external assistance both experiencing terror of co-workers and recovering from the trauma. The forms of individual assistance to the victim, identified in the literature, are presented in Table 2.

**Table 2:** Forms of assistance to the victim of mobbing.

Forms of assistance	Nature of assistance	Source
Social support	Intervention in the mobbing process (stopping the persecution, public intercession, etc.) and various forms of emotional support (listening, sharing experiences, encouragement, etc.).	Farley et al. (2023) and Rossiter and Sochos (2018)
Psychological counselling/treatment	Psychological counselling (also for family members) and/or inpatient assistance. Referral to primary care if somatic symptoms are noticed.	Duffy and Sperry (2007), Ferris (2009), Hillard (2009), Looi et al. (2023), and Pellegrini et al. (2018)
Legal	Component of assistance, manifesting itself by information, counselling and representation in court.	Hillard (2009) and Tatar and Yüksel (2019)

According to Tatar and Yüksel (2019), the opportunity to use social support resources can help people to perceive the need to demand justice and to oppose injustice rather than succumb to helplessness and resignation in the face of oppression. A proper perception of mobbing and of the victim’s situation is also required; therefore, Duffy and Sperry (2007) stress that instead of understanding mobbing as a reversion to a more primitive aggressive state common to all animals, it is necessary to perceive that most often, the person who has been mobbed has received little support, has been isolated, and has constructed a meaning of experience as a shameful personal failure. Victims therefore need support, appropriate treatment and sometimes, vocational rehabilitation.

Since the victim often feels betrayed and abandoned, this loss can be compensated by the attention of the immediate environment: co-workers, family members, friends, etc. It is significant that the support of co-workers and the immediate social setting is necessary both during the entire mobbing process and after it ends, while the help of different specialists can be used depending on the circumstances of a particular case. Meanwhile, psychological help can be effective if the specialist knows what mobbing is and is able to recognise its consequences (Ferris, 2009). This knowledge is also needed for the specialist to find additional sources of assistance, for example, of a lawyer from a professional organisation, etc., (Looi et al., 2023).

## 5 A Christian perspective on spiritual assistance

Christian spiritual help responds to the religious and spiritual needs of persons who have found themselves in difficult situations (Wells et al., 2021) and is grounded on a holistic approach to the person, acknowledging the importance of biological, psychological, religious and social aspects of human existence (Vveinhardt and Deikus, 2022b). This perspective combines the achievements of psychological science and religious resources (spiritual advice, listening, prayer, religious rituals, community support, etc.; Vensel, 2012; Turner, 2018; Hațegan, 2021; Vveinhardt and Deikus, 2022b). Two distinct but intertwining perspectives can be distinguished in such assistance. These perspectives include various crisis coping practices that can be carried out (1) individually by the person with the help of a religious counsellor, and (2) together with the community and its support (Table 3).

**Table 3:** Perspectives on religion-based coping with the crisis.

Perspective	Methods	Key features	Source
Individual	A prayer	Colloquial and meditative styles of prayer help to freely experience unprocessed and difficult emotions without feeling abandoned by God. This allows to effectively cope with grief experiences, leads to gratitude and hopeful disposition, enhances the emotional regulation ability, and provides more opportunities to meaningfully establish supportive and caring relationships.	Jueckstock (2018)
	The Holy Bible	The cross becomes the Gospel of traumatised people only if they see the divine love there, which is willing to bear what mortal, fallen people cannot endure.	Hunsinger (2011)
	Forgiveness	Forgiveness can be an effective response to disaster-related injustices, which promotes resilience.	Worthington et al. (2016)
	Reconciliation	The believer’s confession shapes the narrative about trauma, encompassing perception of the trauma and empowerment to alter memory. The idea is not to forget but to direct memory to a proper perspective that will allow the penitent to move forward.	O’Donnell (2018)
Communal	Community support	Family, friends, neighbours and the community at large can be an extremely important support in times of suffering and help bear each other’s burdens.	Entwistle et al. (2018)
	Rituals	The method of the pastoral community’s care constitutes rituals, psalms and songs, worship and the mystery of the Lord’s Supper.	Hunsinger (2011)
	Intercession	The prayer is a common approach, modelled throughout the Holy Scripture, as a means of intercession, expressing compassion and accepting grace.	Entwistle et al. (2018)
	Homily	Homilies that are based on the whole experience of Jesus, by identifying oneself with His resurrection, open up the possibility of hope for those who are experiencing traumas.	Sanken (2017)
	Humble listening	Those who provide spiritual and emotional care should listen without judging the stories and emotions of those who have experienced them. In doing so, humility is important.	Entwistle et al. (2018)

The overview of religious practices, the effectiveness of which has been confirmed by scientific research, presented in Table 3, shows a broad arsenal of religion-based ways of coping with crisis situations. According to Hunsinger (2011), personal trauma and losses during the liturgy should be empathetically embraced and woven into the losses of a larger community; for this reason, personal suffering turns into part of the communal grief of God’s nation throughout the ages. Rituals, psalms, hymns, worship and participation in the mystery of the Lord’s Supper serve this purpose. Although Christians often associate the adversity with God’s punishment, Bible stories (e.g., Job’s, Jonah’s) teach us to be careful about trying to associate personal suffering with God’s punishment and to be open to any help (Entwistle et al., 2018). Meanwhile, psychoeducation that teaches forgiveness can be useful not only in Christian congregations but also in individual and group psychotherapy, employee assistance programs (Worthington et al., 2016).

Paraphrasing Vensel (2012), religion-based coping describes how persons, based on their faith, behave while attempting to cope with the consequences caused by a situation they have encountered. Research shows that important factors in such coping are faith-based hope and optimism (Sanken, 2017; Prazeres et al., 2021), acceptance and regulation of complex emotions (Entwistle et al., 2018; Jueckstock, 2018; Harris et al., 2021) as well as making sense of painful experiences (Hunsinger, 2011; Sanken, 2017). According to Jacobsen (2017), the context of the Apostles’ Creed in Christian narratives and its Christian statements become the site of transforming the identity determined by trauma, considering the identity of Christ, since such identity is linked to the promise of salvation.

## 6 Discussion and conclusions

This article explored the perspective of integrating Christian spiritual assistance resources in coping with the individual consequences of workplace mobbing. The conducted research review has shown that mobbing is a complex and multifaceted phenomenon that affects not only physical and psychological but also social aspects of human existence. In the scientific literature, three forms of individual assistance to victims of mobbing are usually distinguished (see Table 2), but our review of the literature shows that when helping religious persons who have found themselves in crisis situations, their religious and spiritual needs must also be taken into account. Therefore, for victims of mobbing who identify themselves as Christians, we propose to include a dimension based on Christian spirituality when providing legal aid, social support and counselling/treatment. It is also significant in that religious people who are going through traumatising experiences seek assistance from God and people, which has a positive effect on post-traumatic growth (Harris et al., 2008). In addition, a recent study in which the majority of respondents were Christians demonstrated that the experiences of mobbing were a strong stimulus to seek religious spiritual help (Vveinhardt and Deikus, 2023). Therefore, considering the research results, Christian spiritual assistance can be meaningful in two ways: (a) performing an auxiliary function and (b) performing a direct function in a certain limited scope related to satisfying religious spiritual needs. Auxiliary and direct functions mean the recognition of competences in a certain field and collaboration. Providers of social support (e.g., trade unions, victim support groups), psychological assistance/treatment and legal services should recognize the specific needs of victims and collaborate with representatives of the religious community (clergy, chaplains, religious counsellors). According to Prout et al. (2021), Christians who consider themselves particularly religious prefer to discuss their difficulties in a counseling environment that affirms their Christian faith. In this case, Christian spiritual assistance performs an auxiliary function, and in order to recognize and satisfy the spiritual needs of clients, lay professionals particularly need religious and spiritual literacy (Maximo, 2019). Meanwhile, the direct function is related to religious rituals, spiritual practices, religious counseling, etc. That is, practices that require specific religious preparation or consecration (Table 4).

**Table 4:** Integrating Christian spiritual assistance in overcoming the consequences of mobbing.

Directions for help	Christian spiritual assistance	
	Auxiliary function	Direct function
Social support	Recognition of specific religious spiritual needs that can be met by the religious community in secular aid organizations.	Community support for the victim of mobbing and his / her family members (rituals, intercessory prayer, listening, homily, etc.), integrating it into the community’s religious and social life.
Psychological counselling/treatment	Recognition of religious spiritual needs in psychological counselling/treatment. Collaboration with a clergyman or spiritual counsellor.	Counselling using religious resources (prayer, the Holy Bible, etc.). Religious meaning-making of mobbing experience. Guidance towards forgiveness and reconciliation. Referral to health care professionals.
Legal	Support during and after the process.	Referral to a legal assistance specialist.

*Social support*. It should be noted that both providing psychological or medical assistance and when the religious counsellor is involved in assistance provision, community participation remains equally important. According to Drescher and Foy (2010), when the pastoral counsellor acts together with the community, such personal needs as physical and psychological safety, which the victim and his family experience as members of the church community, are met, excessive sensitivity decreases, learning to trust and build social relationships takes place, and a sense of meaning is discovered in serving others. In other words, authors identify problems of persons who have gone through traumatising psychological and social experiences, which are similar to those of the victims of mobbing (*cf.* Table 1). In addition, Doehring (2019) points out that, in the context of the experienced trauma, religious rituals performed by the community improve spiritual integrity, integration of individuals, families and the community, and promote flexible ways to share suffering, meanings and sources of hope. This means that in practice, lay counselors or public organizations helping victims of mobbing should consider the value of the support provided by the religious community. That is, alongside secular assistance, the support that the Christian community can provide will perform an auxiliary function. Meanwhile, it falls to the leaders of Christian communities to perform the task of preparing the community to be ready to fulfill its direct function and to accept the vulnerable person (as well as the family members who feel the consequences) with specific experiences caused by mobbing. It is important that if secular help is limited in time (while the problem is being addressed), the social relations created in the community do not have such limitation and are related to the very essence of the Church.

*Psychological counselling/treatment*. According to Larson and Larson (2003), scientific research confirms the importance of active spirituality or religiosity as a potential health factor, since it can provide resources for coping with suffering, improve pain management and treatment results. In the first case (auxiliary function), it makes sense in psychotherapy to consider the victim’s religiosity as an additional factor facilitating recovery. The benefit of such recognition of religious spiritual needs in psychotherapy is supported by the results of a meta-analysis of research, conducted by Captari et al. (2018). They show that psychotherapy tailored to clients’ religious and spiritual values resulted in greater improvement in clients’ psychological and spiritual functioning, protection against depression, and reduced risk of substance abuse and suicide. In addition, it has been found that forgiveness is an effective response to disaster-related injustices, which promotes resilience (Worthington et al., 2016). Because specific religious knowledge may be required, it may be helpful to collaborate with a religious counsellor in this case, either through counselling or referral to a clergyman. That is, the counsellor must be prepared to make an appropriate referral to a spiritual healing specialist who would correspond to the theology of the victim (Vensel, 2012). For example, dealing with the issues of forgiveness or reconciliation may require an authoritative theological explanation or a sacramental celebration of reconciliation. Forgiving the wrongdoer is extremely difficult, but the Gospels contain inspiring examples that help us to understand that forgiveness comes from the fact that every person enjoys God’s mercy, which he or she extends in his relationships with others (*cf.* Matthew 18: 21–35), and forgiveness is possible in the most radical situations (*cf.* Luke 23:24). This can be applied through Lectio divina (reading, reflection, prayer, contemplation) or other methods. On the other hand, although religious counsellors commonly use general counselling techniques, grounding on the theories of one or several schools of psychotherapy (Billings, 2000), specific treatment may be required, which can be provided only by a specialist of a particular field.

*Legal assistance.* Mobbing cases usually require decisions that require legal knowledge (disciplinary sanctions, dismissal, compensation for damages, etc.). The religious counsellor should be prepared to refer to a legal professional and simultaneously provide spiritual support throughout the process or even after it has ended. Thus, not only openness to the needs of the victim of mobbing, flexibility, but also specific knowledge is required: for the psychotherapist, about the possibilities of religious spiritual assistance, and for the spiritual assistant, about the process of mobbing and its consequences for the person.

This study has several limitations. We have focused exclusively on the spiritual resources of the Christian religion and shown the possibility of integrating them. In other words, Christianity is used as one of many possible examples; therefore, other studies should additionally examine the spiritual resources of other monotheistic and polytheistic religions, not leaving out non-religious spirituality as well. In addition, the inclusion of other databases could also increase the reviews sample. Also, this article did not examine the specific religious needs of victims of mobbing and methods of religious assistance, which could be the subject of further research.

## Author contributions

JV: Conceptualization, Formal analysis, Funding acquisition, Project administration, Supervision, Writing – original draft, Writing – review & editing. MD: Conceptualization, Formal analysis, Resources, Writing – original draft.
